# Association between Hematocrit and Acute Kidney Injury in Patients with Acute Myocardial Infarction

**DOI:** 10.31083/j.rcm2506228

**Published:** 2024-06-24

**Authors:** Sheng Su, Likun Zhou, Le Li, Zhuxin Zhang, Yulong Xiong, Zhenhao Zhang, Zhao Hu, Yan Yao

**Affiliations:** ^1^Chinese Academy of Medical Sciences, Peking Union Medical College, National Center for Cardiovascular Diseases, Fuwai Hospital, 100037 Beijing, China

**Keywords:** acute myocardial infarction, acute kidney injury, hematocrit, logistic regression, marginal effect

## Abstract

**Backgrounds:**

Hematocrit is found an independent risk factor for acute kidney injury (AKI) in certain patients, but this effect in patients with acute myocardial infarction (AMI) is unclear. We aim to identify the relationship between hematocrit and AKI in patients with AMI.

**Methods:**

The patient data for the discovery and validation cohorts were extracted from the electronic Intensive Care Unit database and the Medical Information Mart for Intensive Care III database, respectively, to identify the relationship between hematocrit and AKI. With normal hematocrit as the reference, patients were divided into five groups based on the initial hematocrit value. The primary outcome was AKI during hospitalization. A multivariable logistic regression and a marginal effect analysis were used to evaluate the relationship between hematocrit and AKI.

**Results:**

In this study, a total of 9692 patients diagnosed with AMI were included, with 7712 patients in the discovery cohort and 1980 patients in the validation cohort. In the discovery cohort, hematocrit in 30–33%, 27–30% or <27% were independent risk factors for AKI in the multivariate logistic analysis, with odds ratio (OR) of 1.774 (95% confidence interval [CI]: 1.203–2.617, *p* = 0.004), 1.834 (95% CI: 1.136–2.961, *p* = 0.013) and 2.577 (95% CI: 1.510–4.397, *p* < 0.001), respectively. Additionally, in the validation cohort, low hematocrit levels independently contributed to an increased risk of AKI among patients with AMI. During the analysis of marginal effects, a significant negative linear relationship between hematocrit levels and AKI was observed.

**Conclusions:**

Decreased hematocrit was an independent risk factor for AKI in patients with AMI. The relationship between hematocrit and AKI was negative linear.

## 1. Introduction

Acute kidney injury (AKI) frequently arises as a complication during the hospitalization of patients experiencing acute myocardial infarction (AMI) [1]. Numerous studies have elucidated a robust correlation between cardiac and renal function, underscoring AKI as an independent risk factor for mortality in AMI patients [2, 3, 4]. Regrettably, as of now, no established therapeutic interventions have demonstrated efficacy in improving outcomes for patients afflicted with AKI [5]. While renal replacement therapy (RRT) serves as a crucial intervention for severe AKI, its application is hindered by potentially life-threatening complications and limited accessibility in certain regions and healthcare settings [6]. Consequently, there is an imperative need to identify opportunities for preventing AKI within this clinical context.

Hematocrit (HCT) is a vital parameter that quantifies the percentage of red blood cells in the total blood volume, which plays a pivotal role in determining blood viscosity and regulating blood flow [7]. Previous investigations have elucidated an inverse correlation between HCT levels and the incidence of AKI among patients undergoing on-pump cardiac surgery [8, 9, 10]. Moreover, a reduced HCT has been recognized as an independent predictor of deterioration in renal function [11]. However, the association between HCT and AKI in individuals with AMI remains unexplored. The objective of this study was to assess whether reduced HCT was associated with an increased risk of AKI in patients with AMI.

## 2. Materials and Methods

### 2.1 Source of Data

The discovery cohort for our study consisted of patients selected from the electronic Intensive Care Unit Collaborative Research Database (eICU, version 2.0), which is a comprehensive database containing detailed information from multiple intensive care units (ICUs). The database includes data from over 200,000 ICU admissions that took place between 2014 and 2015 in 208 hospitals across the United States [12]. The validation cohort data were obtained from the Medical Information Mart for Intensive Care III (MIMIC-III, version 1.4), which is an extensive database containing the medical records of 60,000 intensive care unit (ICU) patients treated at the Beth Israel Deaconess Medical Center in Boston, Massachusetts, USA, from 2001 to 2012 [13]. In the context of routine clinical activities, all data are systematically acquired by computerized means, obviating the direct involvement of medical personnel in the data collection process. One author (LL) completed a web course titled “Protecting Human Research Subjects” offered by the National Institutes of Health, and obtained the corresponding certificate (ID: 35965741). Possessing the requisite qualifications, LL is duly authorized to initiate inquiries into the information repositories. It is noteworthy that the establishment of the databases received explicit approval from the Massachusetts Institute of Technology, granted under an exemption for informed consent.

### 2.2 Study Population

We analyzed data from consecutive patients aged 18 years or older who were diagnosed with AMI. All patients received Guideline-Directed Medical Therapy. The definition of AMI was based on the clinical practice guideline [14]. AKI was diagnosed using the criteria proposed by Kidney Disease: Improving Global Outcomes. These criteria include an increase in serum creatinine (SCr) by ≥0.3 mg/dL (or ≥26.5 µmol/L) within 48 hours, an increase in SCr to 1.5 times the baseline level within 7 days, and a urine output (UO) of ≤0.5 mL/kg/h for 6 hours [15]. 963 (11.1%) patients were excluded due to lack of data.

### 2.3 Data Collection

Patients’ data, including demographics (age, gender, race, weight), vital signs (temperature, blood pressure, heart rate, respiratory rate, UO), common comorbidities (congestive heart failure, hypertension, diabetes, chronic pulmonary obstructive disease, sepsis, renal disfunction, hepatic disfunction, stroke, cancer), and laboratory tests results (white blood cell count, red blood cell count, HCT, hemoglobin, platelet count, serum sodium, serum potassium, serum calcium, serum chloride, serum glucose, serum creatine kinase, serum aniongap, serum bicarbonate, SCr, blood urine nitrogen) were included in the initial analysis. In the two public databases, for patients with multiple ICU admissions, only the data from the first ICU admission were selected for analysis.

### 2.4 Primary Exposure Variable and Outcomes

The principal determinant examined in this analysis was the initial HCT level upon hospitalization. The categorization of HCT was performed in accordance with the World Health Organization’s definition of anemia [16], stratifying it into five groups: (1) normal range (HCT ≥36% in females and ≥39% in males), (2) mild reduction (33% ≤ HCT < 36% in females or 33% ≤ HCT < 39% in males), (3) 30% ≤ HCT < 33%, (4) 27% ≤ HCT < 30%, and finally, (5) HCT <27%. Previous research has posited that male patients exhibit a heightened susceptibility to AKI in comparison to their female counterparts when confronted with a decline in HCT levels [9]. In order to mitigate potential sex stratification effects, a subgroup analysis stratified by gender was performed.

We aimed to identify the relationship between HCT and renal dysfunction in patients with AMI. The main outcome of interest was AKI during hospitalization. The secondary outcomes included RRT and hospital mortality.

### 2.5 Statistical Analyses

The assessment of data normal distribution was conducted utilizing the Kolmogorov-Smirnov test. Continuous variables were presented as mean ± standard deviation (SD) and subjected to comparison through the *t*-test. Levene’s test was used to assess the assumption of homogeneity of variance. When the assumption of homoscedasticity was violated, intergroup comparisons were conducted using the Welch’s *t*-test. Proportions were used to represent categorical data, and the chi-squared test was employed for their analysis. Multiple imputation techniques were employed to address missing variables [17].

Multivariable logistic regression analysis employing stepwise backward selection methodology was employed for covariate adjustment, incorporating variables identified to exhibit marginal association (*p* < 0.10) on univariate analysis with AKI. Adjusted risk estimates were obtained by constructing odds ratios (OR) along with 95% confidence intervals (CI). The marginal effects of HCT in predicting AKI were investigated. In addition, an evaluation of the variance inflation factor (VIF) among the covariates in a logistic model was used to assess potential multicollinearity. Variables demonstrating a VIF >4.0, indicative of multicollinearity, were excluded in the multivariate logistic regression analysis. Statistical significance was defined as *p*-values less than 0.05 (two-tailed). All statistical analyses were conducted using Stata version 15.0 (StataCorp, College Station, TX, USA) and R software version 4.0.4 (R Foundation for Statistical Computing, Vienna, Austria).

## 3. Results

### 3.1 Baseline Characteristics

The discovery cohort consisted of a total of 7712 AMI patients. Among them, 950 patients (12.3%) experienced AKI during hospitalization (**Supplementary Table 1**). In short, mean age was 65.0 ± 12.6 years, 4924 (63.8%) were men, and 3944 (51.1%) were diagnosed as ST-elevation myocardial infarction. AKI group compared to non-AKI group were older (68.4 ± 12.5 *vs*. 64.5 ± 12.6, *p* < 0.001) and had significantly lower HCT (36.4 ± 7.8 *vs*. 39.5 ± 6.5, *p* < 0.001). We identified 17 variables using stepwise backward approach to build logistic regression model in the discovery cohort, and only data of the 17 variables of the validation cohort were extracted for convenience. In the validation cohort, a total of 1980 patients with AMI were included, 279 patients (14.1%) were diagnosed as AKI and patients with AKI were found to be older compared to those without AKI (72.3 ± 12.9 *vs*. 67.1 ± 14.2, *p* < 0.001). The AKI group exhibited lower HCT levels compared to the non-AKI group (31.7 ± 4.4 *vs*. 33.7 ± 4.8, *p* < 0.001). Table 1 presents additional comparisons between the non-AKI and AKI groups in both the discovery and validation cohorts.

**Table 1.** **Baseline characteristic of the cohorts**.

Variables		Discovery cohort (n = 7712)			Validation cohort (n = 1980)		
		AKI	Non-AKI	*p* value	AKI	Non-AKI	*p* value
		(n = 950)	(n = 6762)		(n = 279)	(n = 1701)	
Age, years		68.4 ± 12.5	64.5 ± 12.6	<0.001	72.3 ± 12.9	67.1 ± 14.2	<0.001
UO, mL/kg/h		0.87 ± 0.80	1.13 ± 0.95	<0.001	0.87 ± 0.67	1.18 ± 0.73	<0.001
RR, bpm		21 ± 6	19 ± 6	<0.001	20 ± 4	18 ± 3	<0.001
MAP, mmHg		76.9 ± 10.7	78.5 ± 10.1	<0.001	74.6 ± 9.8	77.8 ± 10.1	<0.001
Heart rate, bpm		94 ± 21	85 ± 19	<0.001	88 ± 20	84 ± 17	<0.001
${\mathrm{SpO}}_{2}$, %		96.4 ± 5.2	97.0 ± 3.9	<0.001	96.8 ± 3.2	97.2 ± 2.8	0.028
AMI							
	STEMI	266 (28.0)	3678 (54.4)	<0.001	105 (37.5)	899 (52.8)	<0.001
	NSTEMI	684 (72.0)	3084 (45.6)	<0.001	174 (62.5)	802 (47.2)	<0.001
Invasive strategy							
	PTCA + CAG	201 (21.2)	3251 (48.1)	<0.001	89 (31.9)	755 (44.4)	<0.001
	CAG	116 (12.2)	1310 (19.4)	<0.001	31 (11.1)	336 (19.8)	<0.001
CHF, %		264 (27.8)	685 (10.1)	<0.001	158 (56.6)	558 (32.8)	<0.001
VA, %		79 (8.3)	319 (4.7)	<0.001	42 (15.1)	299 (17.6)	0.301
COPD, %		111 (11.7)	357 (5.3)	<0.001	41 (14.7)	182 (10.7)	0.050
Sepsis, %		328 (34.5)	419 (6.2)	<0.001	52 (18.9)	83 (4.9)	<0.001
CKD, %		215 (22.6)	552 (8.2)	<0.001	40 (14.3)	70 (4.1)	<0.001
Anemia, %		432 (45.5)	1336 (19.7)	<0.001	111 (39.8)	392 (23.0)	<0.001
WBC, × ${\mathrm{10}}^{9}$/L		14.2 ± 7.8	11.6 ± 6.5	<0.001	13.8 ± 6.6	12.4 ± 5.3	<0.001
Potassium, mmol/L		4.52 ± 0.97	4.06 ± 0.61	<0.001	4.33 ± 0.80	4.19 ± 0.71	0.006
SCr, mg/dL		2.40 ± 1.49	1.27 ± 1.02	<0.001	1.89 ± 1.23	1.16 ± 1.14	<0.001
BUN, mg/dL		41.3 ± 23.4	21.1 ± 13.2	<0.001	39.5 ± 24.1	21.3 ± 14.5	<0.001
Hematocrit, %		36.4 ± 7.8	39.5 ± 6.5	<0.001	31.7 ± 4.4	33.7 ± 4.8	<0.001
	HCT <27	109 (11.5)	270 (4.0)		47 (16.8)	164 (9.6)	
	27 ≤ HCT < 30	89 (9.4)	270 (4.0)		45 (16.1)	185 (10.9)	
	30 ≤ HCT < 33	123 (12.9)	457 (6.8)		44 (15.8)	250 (14.7)	
	Mild reduction	217 (22.8)	1240 (18.3)		82 (29.4)	268 (33.4)	
	Normal	412 (43.3)	4525 (66.9)	<0.001*	61 (21.9)	534 (31.4)	<0.001*

AKI, acute kidney injury; UO, urine output; RR, respiratory rate; MAP, mean aortic pressure; ${\mathrm{SpO}}_{2}$, saturation of pulse oxygen; AMI, acute myocardial infarction; STEMI, ST-segment elevation myocardial infarction; NSTEMI, Non-ST-segment elevation myocardial infarction; PTCA, percutaneous transluminal coronary angioplasty; CAG, coronary angiography; CHF, congestive heart failure; VA, ventricular arrhythmia; COPD, chronic obstructive pulmonary disease; CKD, chronic kidney disease; WBC, white blood cell; SCr, serum creatinine; BUN, blood urea nitrogen; HCT, hematocrit; Mild reduction, 33% ≤ HCT < 36% in females or 33% ≤ HCT < 39% in males; Normal, HCT ≥36% in females and ≥39% in males; *, *p* for trend.

### 3.2 Primary Outcomes

A multivariable logistic regression analysis was conducted to investigate the correlation between HCT and AKI. In the unadjusted model, low HCT was found to be significantly associated with an increased risk of AKI in AMI patients, with OR of 1.876 (95% CI: 1.572–2.238, *p* < 0.001), 2.933 (95% CI: 2.346–3.665, *p* < 0.001), 3.592 (95% CI: 2.770–4.657, *p* < 0.001) and 4.399 (95% CI: 3.446–5.615, *p* < 0.001) for HCT in mild reduction, 30–33%, 27–30% and <27% groups, respectively, with normal HCT as the reference. Furthermore, 17 variables were incorporated into the covariate adjustment of the univariate logistic analysis (**Supplementary Table 2**). HCT in 30–33%, 27–30% or <27% were identified as independent risk factors for AKI in the model adjusted by the 17 variables, with OR of 1.739 (95% CI: 1.178–2.566, *p* = 0.005), 1.802 (95% CI: 1.115–2.911, *p* = 0.016) and 2.502 (95% CI: 1.467–4.266, *p* < 0.001), respectively. In the validation cohort, we also found that low HCT was an independent risk factor for AKI in AMI patients in the multivariate logistic model, with OR of 1.660 (95% CI: 1.051–2.618, *p* = 0.030) and 1.613 (95% CI: 1.022–2.552, *p* = 0.039) for HCT in 27–30% or <27% respectively (Table 2). To assess the presence of multicollinearity among the 17 variables, a VIF test was performed. All the VIF values were below 4.0, indicating that there was no significant multicollinearity. The average VIF values in the discovery and validation cohorts were 1.49 and 1.16, respectively.

**Table 2.** **Primary outcomes**.

Variables		Non-adjusted		Model 1		Model 2	
		OR	*p* value	OR	*p* value	OR	*p* value
Discovery cohort							
	Normal	Ref.		Ref.		Ref.	
	HCT <27	4.399 (3.446–5.615)	<0.001	2.828 (2.088–3.828)	<0.001	2.502 (1.467–4.266)	0.001
	27 ≤ HCT < 30	3.592 (2.770–4.657)	<0.001	2.051 (1.460–2.880)	<0.001	1.802 (1.115–2.911)	0.016
	30 ≤ HCT < 33	2.933 (2.346–3.665)	<0.001	2.023 (1.527–2.680)	<0.001	1.739 (1.178–2.566)	0.005
	Mild reduction	1.876 (1.572–2.238)	<0.001	1.484 (1.192–1.848)	<0.001	1.334 (0.998–1.783)	0.051
Validation cohort							
	Normal	Ref.		Ref.		Ref.	
	HCT <27	2.509 (1.651–3.813)	<0.001	1.843 (1.182–2.873)	0.007	1.613 (1.022–2.552)	0.039
	27 ≤ HCT < 30	2.129 (1.399–3.240)	<0.001	1.818 (1.173–2.818)	0.007	1.660 (1.051–2.618)	0.030
	30 ≤ HCT < 33	1.541 (1.017–2.335)	0.042	1.120 (0.771–1.867)	0.420	1.078 (0.654–1.802)	0.699
	Mild reduction	1.263 (0.889–1.797)	0.192	1.240 (0.862–1.785)	0.246	1.182 (0.782–1.767)	0.401

Model 1: age, respiratory rate, mean aortic pressure, heart rate, saturation of pulse oxygen; Model 2: age, urine output, respiratory rate, mean aortic pressure, heart rate, saturation of pulse oxygen, ventricular arrhythmia, chronic obstructive pulmonary disease, sepsis, chronic kidney disease, anemia, white blood cell, potassium, creatinine, blood urea nitrogen, coronary angiography. OR, odds ratios; HCT, hematocrit.

**Fig. 1.**
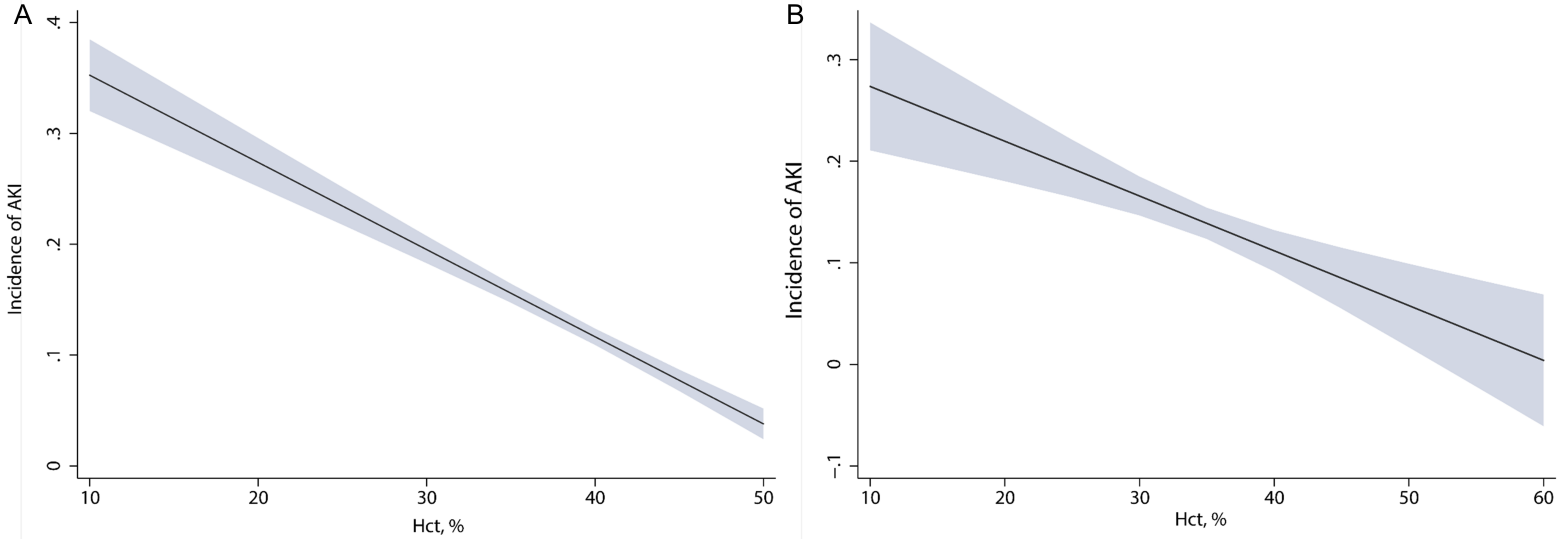
**Relationship between initial HCT and risk of AKI evaluated by marginal effect analysis in patients with AMI.** (A) Marginal effect in the discovery cohort. (B) Marginal effect in the validation cohort. HCT, hematocrit; AKI, acute kidney injury; AMI, acute myocardial infarction.

The marginal effect analysis was employed to evaluate the association between HCT and AKI. Our findings revealed a clear negative linear correlation between HCT and AKI (Fig. 1A). With an increase in the initial HCT upon ICU admission, there was a linear decline in the probability of AKI. In the validation cohort, the linear relationship between HCT and AKI was also found in the marginal effect analysis (Fig. 1B).

### 3.3 Secondary Outcomes

As shown in **Supplementary Fig. 1**, after adjusting for the 17 variables in the logistic model, low HCT was found to be significantly associated with hospital mortality. The OR for HCT between 27–30% and below 27% were 1.557 (95% CI: 1.007–2.407, *p* = 0.047) and 1.755 (95% CI: 1.169–2.634, *p* = 0.007), respectively. Furthermore, in the multivariate logistic model, there was a significant association between low HCT and the need for RRT during hospitalization. The OR for HCT levels in mild reduction, 30–33%, 27–30%, and below 27% were 2.353 (95% CI: 1.510–3.670, *p* < 0.001), 3.159 (95% CI: 1.878–5.312, *p* < 0.001), 3.575 (95% CI: 1.991–26.418, *p* < 0.001), and 3.800 (95% CI: 2.182–6.618, *p* < 0.001), respectively (**Supplementary Fig. 2**).

**Fig. 2.**
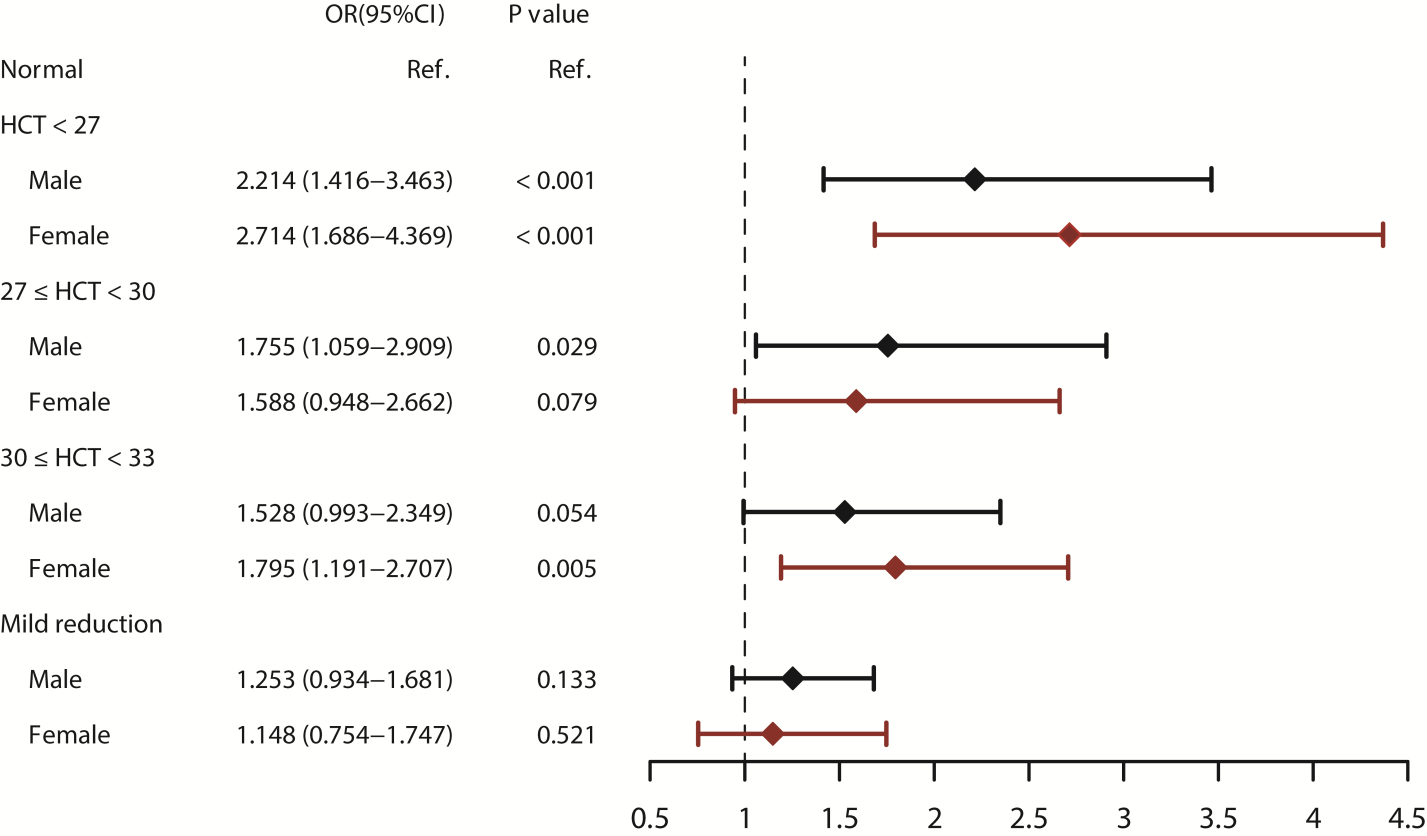
**Subgroup analysis of sex on the relationship between HCT and AKI assessed by the multivariate logistic regression.** HCT, hematocrit; AKI, acute kidney injury.

In order to reduce a possible sex stratification effect, we performed a subgroup analysis separated by sex. After adjusting for other variables in the multivariate logistic model, HCT below 27% was independently associated with an increased risk of AKI in both male and female patients. The OR in male patients was 2.214 (95% CI: 1.416–3.463, *p* < 0.001), while in female patients, the OR was 2.714 (95% CI: 1.686–4.369, *p* < 0.001) (Fig. 2). The predictive marginal effect analysis showed a linear relationship between HCT and AKI in both male and female patients (**Supplementary Fig. 3**).

## 4. Discussion

This study aimed to investigate the association between HCT and the occurrence of AKI in AMI patients. We found that HCT was negatively associated with AKI, and the correlation was approximately linear. Furthermore, this correlation was consistent across genders, as low HCT was strongly associated with AKI in both male and female patients. Additionally, low HCT was found to be an independent risk factor for RRT during hospitalization and hospital mortality.

HCT, defined as the percentage of blood volume comprised of red blood cells, serves as a pivotal factor influencing various physiological parameters. It significantly contributes to the regulation of whole blood viscosity, blood pressure, venous return, cardiac output, and platelet adhesiveness [18, 19, 20]. As HCT rises, the blood viscosity increases rapidly, and vice versa. Therefore, HCT could be used as a reference for fluid infusion. Additionally, HCT is considered one of the most precise methods of determining the degree of anemia [21].

From a pathophysiological perspective, the etiology of AKI can be categorized into three overarching classifications: prerenal, characterized by diminished renal perfusion; intrinsic renal, stemming from pathological processes within the glomeruli and tubules; and postrenal, resulting from obstructive conditions in the urinary tract [22]. Prerenal AKI is the predominant form globally [22], and is correlated with diminished renal perfusion and glomerular filtration rate (GFR), attributable to intravascular volume depletion stemming from hypovolemia, peripheral vasodilation, reduced arterial pressures, and compromised cardiac function, culminating in a reduction of cardiac output. Patients with AMI are characterized as acute myocardial ischemia which significantly affects cardiac pump function. Therefore, renal hypoperfusion is common in patients with AMI because of a low cardiac output state, which is also called cardiorenal syndrome [23].

Given the above evidence, low HCT could reflect the reduced effective circulatory volume, which significantly reduces renal perfusion and induces prerenal AKI in patients with AMI. Habib *et al*. [24] have demonstrated that HCT of cardiopulmonary bypass <24% was associated with a systematically increased likelihood of AKI. Sukmark *et al*. [25] have found that HCT <30% was independently associated with an increased risk of the worsening renal function in critical ill patients, with OR of 1.81 (95% CI: 1.50–2.19, *p* < 0.001). Mehran *et al*. [26] found that low HCT (baseline HCT <39% for men and <36% for women) was a risk factor for contrast-induced nephropathy in non-AMI patients undergoing percutaneous coronary intervention. Additionally, we found that HCT was remarkably correlated linearly with AKI in AMI patients, which aligned with previous research [9, 10]. Moreover, Paul *et al*. [27] suggested that HCT showed a strong association with an increased risk of cardiovascular mortality. We performed a multivariable logistic analysis to evaluate the relationship between HCT and hospital mortality and found that low HCT was strongly associated with hospital mortality. There are few effective methods to improve outcomes for AKI, and a number of patients with AKI would be treated with RRT. To explore the relationship between HCT and RRT, a multivariable logistic analysis was conducted. The findings revealed a significant and strong association between HCT and RRT, indicating that HCT was highly correlated with the requirement for RRT.

Prior investigations have elucidated a sexual dimorphism in the context of HCT and AKI. Female patients demonstrated a heightened propensity to experience reduced hematocrit levels compared to their male counterparts, consequently manifesting an augmented overall susceptibility to AKI following meticulous adjustment for risk factors [9]. Kang *et al*. [28] attributed this phenomenon to the protective effect of estrogen against ischemic injury. Furthermore, Mehta *et al*. [8] proposed that menstruation, coupled with its concomitant blood loss, may afford women the capacity to optimize oxygen extraction and delivery to tissues even at reduced systemic HCT levels. However, Brescia *et al*. [10] found no sex-related differences in the effect of HCT on AKI in patients who underwent coronary artery bypass grafting. The effect of sex on the relationship between HCT and AKI is still controversial. In the present study, we conducted a subgroup analysis stratified by sex, and the results showed that HCT <27% was an independent risk factor both in female and male patients, the effect of HCT on AKI appeared to be gender-independent. Future prospective studies are needed to address the issue.

## 5. Limitations

While this study represents a comprehensive analysis based on a large-scale cohort and has undergone external validation to substantiate its primary findings, it is essential to acknowledge certain limitations inherent in the current investigation. Firstly, the potential influence of unmeasured confounding factors cannot be definitively excluded. Rigorous methodologies, such as risk adjustment, were employed to address discernible variations in hospital admission characteristics. However, it is imperative to note that the analysis of potential AKI risk factors was confined to the data accessible in the public database. Secondly, due to the presence of missing and extreme data points in the public database, pivotal variables with a notable degree of missing data, including left ventricular ejection fraction and the use of contrast, were regrettably excluded from the analysis. Furthermore, specific imputation methods were applied to address missing data, potentially impacting the robustness of the results. Lastly, it is crucial to recognize the retrospective nature of this cohort study. While external validation provides support for the findings, prospective clinical trials are indispensable to further substantiate and generalize the results. The necessity for prospective investigations is paramount to establish a causal relationship and enhance the clinical applicability of the observed associations.

## 6. Conclusions

HCT was an independent risk factor for AKI and hospital mortality in patients with AMI. The relationship between HCT and AKI was negatively linear, and was gender-independent. In addition, HCT was also found an independent risk factor for RRT in these patients.

## Data Availability

The datasets used during the current study are available from the corresponding author on reasonable request.

## Supplementary Material

Supplementary material associated with this article can be found, in the online version, at https://doi.org/10.31083/j.rcm2506228.

## References

[1] Kaltsas E, Chalikias G, Tziakas D (2018). The Incidence and the Prognostic Impact of Acute Kidney Injury in Acute Myocardial Infarction Patients: Current Preventive Strategies. *Cardiovascular Drugs and Therapy*.

[2] Marenzi G, Cosentino N, Bartorelli AL (2015). Acute kidney injury in patients with acute coronary syndromes. *Heart (British Cardiac Society)*.

[3] Parikh CR, Coca SG, Wang Y, Masoudi FA, Krumholz HM (2008). Long-term prognosis of acute kidney injury after acute myocardial infarction. *Archives of Internal Medicine*.

[4] Chalikias G, Serif L, Kikas P, Thomaidis A, Stakos D, Makrygiannis D (2019). Long-term impact of acute kidney injury on prognosis in patients with acute myocardial infarction. *International Journal of Cardiology*.

[5] Matuszkiewicz-Rowińska J, Małyszko J (2020). Acute kidney injury, its definition, and treatment in adults: guidelines and reality. *Polish Archives of Internal Medicine*.

[6] Vanmassenhove J, Kielstein J, Jörres A, Biesen WV (2017). Management of patients at risk of acute kidney injury. *Lancet (London, England)*.

[7] de Simone G, Devereux RB, Chien S, Alderman MH, Atlas SA, Laragh JH (1990). Relation of blood viscosity to demographic and physiologic variables and to cardiovascular risk factors in apparently normal adults. *Circulation*.

[8] Mehta RH, Castelvecchio S, Ballotta A, Frigiola A, Bossone E, Ranucci M (2013). Association of gender and lowest hematocrit on cardiopulmonary bypass with acute kidney injury and operative mortality in patients undergoing cardiac surgery. *The Annals of Thoracic Surgery*.

[9] Ellis MC, Paugh TA, Dickinson TA, Fuller J, Chores J, Paone G (2015). Nadir Hematocrit on Bypass and Rates of Acute Kidney Injury: Does Sex Matter. *The Annals of Thoracic Surgery*.

[10] Brescia AA, Wu X, Paone G, Heung M, Paugh TA, Shann KG (2019). Effect of sex on nadir hematocrit and rates of acute kidney injury in coronary artery bypass. *The Journal of Thoracic and Cardiovascular Surgery*.

[11] Pourafkari L, Arora P, Porhomayon J, Dosluoglu HH, Arora P, Nader ND (2018). Acute kidney injury after non-cardiovascular surgery: risk factors and impact on development of chronic kidney disease and long-term mortality. *Current Medical Research and Opinion*.

[12] Pollard TJ, Johnson AEW, Raffa JD, Celi LA, Mark RG, Badawi O (2018). The eICU Collaborative Research Database, a freely available multi-center database for critical care research. *Scientific Data*.

[13] Johnson AEW, Pollard TJ, Shen L, Lehman LWH, Feng M, Ghassemi M (2016). MIMIC-III, a freely accessible critical care database. *Scientific Data*.

[14] Thygesen K, Alpert JS, Jaffe AS, Simoons ML, Chaitman BR, White HD (2012). Third universal definition of myocardial infarction. *Journal of the American College of Cardiology*.

[15] Khwaja A (2012). KDIGO clinical practice guidelines for acute kidney injury. *Nephron. Clinical Practice*.

[16] World Health Organization (2011). Haemoglobin concentrations for the diagnosis of anemia and assessment of severity. https://iris.who.int/bitstream/handle/10665/85839/WHO_NMH_NHD_MNM_11.1_eng.pdf?sequence=22.

[17] Zhang Z (2016). Multiple imputation with multivariate imputation by chained equation (MICE) package. *Annals of Translational Medicine*.

[18] Guyton AC, Richardson TQ (1961). Effect of hematocrit on venous return. *Circulation Research*.

[19] Hellem AJ, Borchgrevink CF, Ames SB (1961). The role of red cells in haemostasis: the relation between haematocrit, bleeding time and platelet adhesiveness. *British Journal of Haematology*.

[20] Vázquez BYS, Martini J, Tsai AG, Johnson PC, Cabrales P, Intaglietta M (2010). The variability of blood pressure due to small changes of hematocrit. *American Journal of Physiology. Heart and Circulatory Physiology*.

[21] Goobie SM, Faraoni D, Zurakowski D, DiNardo JA (2016). Association of Preoperative Anemia With Postoperative Mortality in Neonates. *JAMA Pediatrics*.

[22] Levey AS, James MT (2017). Acute Kidney Injury. *Annals of Internal Medicine*.

[23] Di Lullo L, Bellasi A, Russo D, Cozzolino M, Ronco C (2017). Cardiorenal acute kidney injury: Epidemiology, presentation, causes, pathophysiology and treatment. *International Journal of Cardiology*.

[24] Habib RH, Zacharias A, Schwann TA, Riordan CJ, Engoren M, Durham SJ (2005). Role of hemodilutional anemia and transfusion during cardiopulmonary bypass in renal injury after coronary revascularization: implications on operative outcome. *Critical Care Medicine*.

[25] Sukmark T, Lumlertgul N, Praditpornsilpa K, Tungsanga K, Eiam-Ong S, Srisawat N (2020). SEA-MAKE score as a tool for predicting major adverse kidney events in critically ill patients with acute kidney injury: results from the SEA-AKI study. *Annals of Intensive Care*.

[26] Mehran R, Aymong ED, Nikolsky E, Lasic Z, Iakovou I, Fahy M (2004). A simple risk score for prediction of contrast-induced nephropathy after percutaneous coronary intervention: development and initial validation. *Journal of the American College of Cardiology*.

[27] Paul L, Jeemon P, Hewitt J, McCallum L, Higgins P, Walters M (2012). Hematocrit predicts long-term mortality in a nonlinear and sex-specific manner in hypertensive adults. *Hyertension (Dallas, Tex.: 1979)*.

[28] Kang KP, Lee JE, Lee AS, Jung YJ, Kim D, Lee S (2014). Effect of gender differences on the regulation of renal ischemia-reperfusion-induced inflammation in mice. *Molecular Medicine Reports*.

