# Supplementary material for: Association between Hematocrit and Acute Kidney Injury in Patients with Acute Myocardial Infarction
Source: Rev Cardiovasc Med. 2024 Jun 24;25(6):228. doi: 10.31083/j.rcm2506228 (PMC11270070; doi:10.31083/j.rcm2506228)
Supplement: Supplementary file 1 [file 2153-8174-25-6-228-s1.docx]

| Supplementary Table 1. Comparisons of demographics between AKI and non-AKI. | | | | |
| --- | --- | --- | --- | --- |
| Variables | Total (n = 7712) | AKI (n = 950) | Non-AKI (n = 6762) | *P* value |
| Demographics and characteristics |  |  |  |  |
| Age, years | 65.0 ± 12.6 | 68.4 ± 12.5 | 64.5 ± 12.6 | < 0.001 |
| Male, % | 4924 (63.8) | 578 (60.8) | 4346 (64.3) | 0.039 |
| Weight, kg, | 85.5 ± 21.8 | 85.5 ± 24.9 | 85.5 ± 21.3 | 0.936 |
| STEMI, % | 3944 (51.1) | 262 (27.6) | 3682 (54.5) | < 0.001 |
| UO (first 24-hour), mL/kg/h | 1.09 ± 0.92 | 0.87 ± 0.80 | 1.13 ± 0.95 | < 0.001 |
| Vital signs |  |  |  |  |
| RR, bpm | 19.4 ± 6.2 | 21.1 ± 6.4 | 19.2 ± 6.2 | < 0.001 |
| MAP, mmHg | 77.6 ± 10.5 | 76.9 ± 10.7 | 78.5 ± 10.1 | < 0.001 |
| Heart rate, bpm | 85.8 ± 19.4 | 94.2 ± 21.2 | 84.5 ± 18.8 | < 0.001 |
| SpO_2_, % | 96.9 ± 4.2 | 96.4 ± 5.2 | 97.0 ± 3.9 | < 0.001 |
| Comorbidities |  |  |  |  |
| CHF, % | 949 (12.3) | 264 (27.8) | 685 (10.1) | <0.001 |
| VA, % | 398 (5.2) | 79 (8.3) | 319 (4.7) | < 0.001 |
| COPD, % | 468 (6.1) | 111 (11.7) | 357 (5.3) | < 0.001 |
| Sepsis, % | 747 (9.7) | 328 (34.5) | 419 (6.2) | < 0.001 |
| CKD, % | 767 (10.0) | 215 (22.6) | 552 (8.2) | < 0.001 |
| Anemia, % | 1768 (22.9) | 432 (45.5) | 1336 (19.7) | < 0.001 |
| Laboratory tests |  |  |  |  |
| WBC, × 10^9/L | 11.9 ± 6.7 | 14.2 ± 7.8 | 11.6 ± 6.5 | < 0.001 |
| Platelet, × 10^9/L | 232.9 ± 82.3 | 234.3 ± 80.2 | 222.9 ± 95.0 | < 0.001 |
| RBC, × 10^12/L | 4.36 ± 0.77 | 4.03 ± 0.86 | 4.41 ± 0.75 | < 0.001 |
| Sodium, mmol/L | 137.6 ± 4.3 | 137.8 ± 6.4 | 137.6 ± 3.9 | 0.212 |
| Potassium, mmol/L | 4.12 ± 0.68 | 4.52 ± 0.97 | 4.06 ± 0.61 | < 0.001 |
| Creatinine, mg/dL | 1.41 ± 1.15 | 2.40 ± 1.49 | 1.27 ± 1.02 | < 0.001 |
| BUN, mg/dL | 23.6 ± 16.3 | 41.3 ± 23.4 | 21.1 ± 13.2 | < 0.001 |
| Hematocrit, % | 39.2 ± 6.7 | 36.4 ± 7.8 | 39.5 ± 6.5 | < 0.001 |
| HCT < 27 | 379 (4.9) | 109 (11.5) | 270 (4.0) |  |
| 27 ≤ HCT < 30 | 359 (4.6) | 89 (9.4) | 270 (4.0) |  |
| 30 ≤ HCT < 33 | 580 (7.5) | 123 (12.9) | 457 (6.8) |  |
| Mild reduction | 1457 (18.9) | 217 (22.8) | 1240 (18.3) |  |
| Normal | 4937 (64.0) | 412 (43.3) | 4525 (66.9) | < 0.001* |

AKI: acute kidney injury; STEMI: ST-elevation myocardial infarction; UO: urine output; RR: respiratory rate; MAP: mean aortic pressure; SpO_2_: saturation of pulse oxygen; CHF: congestive heart failure; VA: ventricular arrhythmia; COPD: chronic obstructive pulmonary disease; CKD: chronic kidney injury; WBC: white blood cell; RBC: red blood cell; BUN: blood urea nitrogen; HCT: hematocrit. *:P for trend.

OR: odds ratio; CI: confidence interval; other abbreviations are as same in Table S1.

| Supplementary Table 2. Univariate and multivariate logistic regression analyses for acute kidney injury. | | | | | | | |
| --- | --- | --- | --- | --- | --- | --- | --- |
|  | Univariate logistic analysis | |  | | Multivariate logistic analysis | | |
| Variables | OR (95% CI) | P value | |  | | OR (95% CI) | P value |
| Age | 1.025 (1.019–1.031) | < 0.001 | |  | | 1.011 (1.003–1.019) | 0.006 |
| UO | 0.536 (0.492–0.584) | < 0.001 | |  | | 0.794 (0.679–0.928) | 0.004 |
| RR | 1.044 (1.032–1.056) | < 0.001 | |  | | 1.010 (0.995–1.025) | 0.175 |
| MAP | 0.980 (0.976–0.984) | < 0.001 | |  | | 0.992 (0.987–0.997) | 0.001 |
| Heart rate | 1.023 (1.019–1.027) | < 0.001 | |  | | 1.011 (1.006–1.016) | < 0.001 |
| SpO_2_ | 0.975 (0.961–0.990) | < 0.001 | |  | | 0.993 (0.973–1.012) | 0.448 |
| PTCA | 2.823 (1.701–4.688) | < 0.001 | |  | | 1.605 (0.863–2.985) | 0.135 |
| CAG | 3.450 (2.931–4.061) | < 0.001 | |  | | 1.816 (1.458–2.261) | < 0.001 |
| CHF | 3.367 (2.862–3.962) | < 0.001 | |  | | 1.761 (1.398–2.219) | < 0.001 |
| VA | 1.863 (1.444–2.405) | < 0.001 | |  | | 2.344 (1.651–3.328) | < 0.001 |
| COPD | 2.404 (1.921–3.009) | < 0.001 | |  | | 1.182 (0.867–1.612) | 0.289 |
| Sepsis | 7.756 (6.567–9.160) | < 0.001 | |  | | 3.969 (3.143–5.013) | < 0.001 |
| CKD | 3.397 (2.853–4.045) | < 0.001 | |  | | 1.760 (1.360–2.277) | < 0.001 |
| Anemia | 3.458 (3.003–3.981) | < 0.001 | |  | | 1.873 (1.414–2.479) | < 0.001 |
| WBC | 2.868 (2.264–3.634) | < 0.001 | |  | | 1.015 (1.002–1.027) | 0.025 |
| Potassium | 1.822 (1.594–2.083) | < 0.001 | |  | | 1.825 (1.619–2.056) | < 0.001 |
| Creatinine | 2.053 (1.944–2.168) | < 0.001 | |  | | 1.993 (1.824–2.178) | < 0.001 |
| BUN | 1.051 (1.048–1.054) | < 0.001 | |  | | 1.046 (1.040–1.052) | < 0.001 |


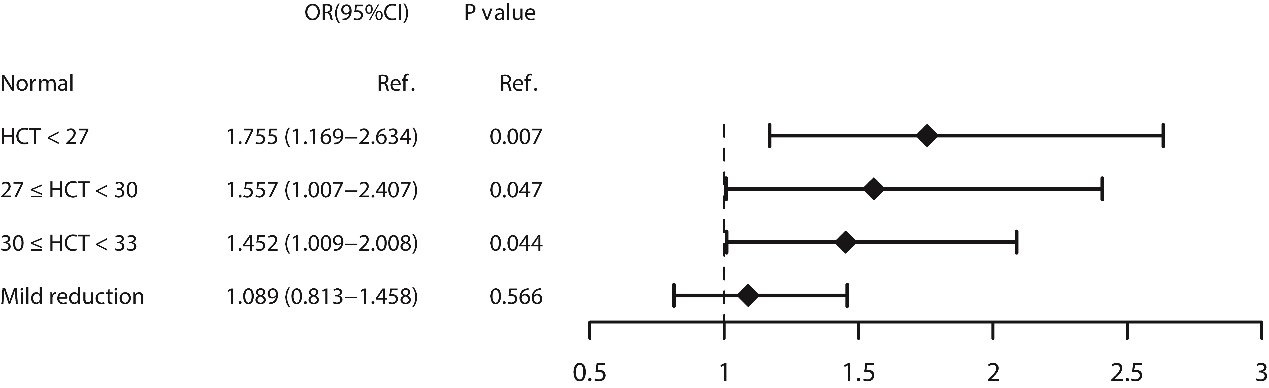


**Supplementary Fig. 1**. Calculated odds ratios for hospital mortality in the multivariate logistic regression.


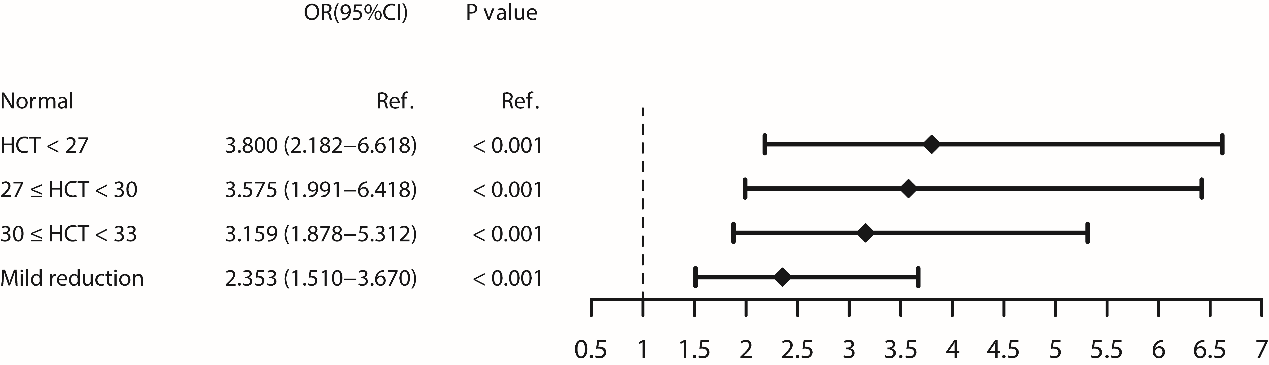


**Supplementary Fig. 2.** Calculated odds ratios for renal replacement therapy in the multivariate logistic regression.


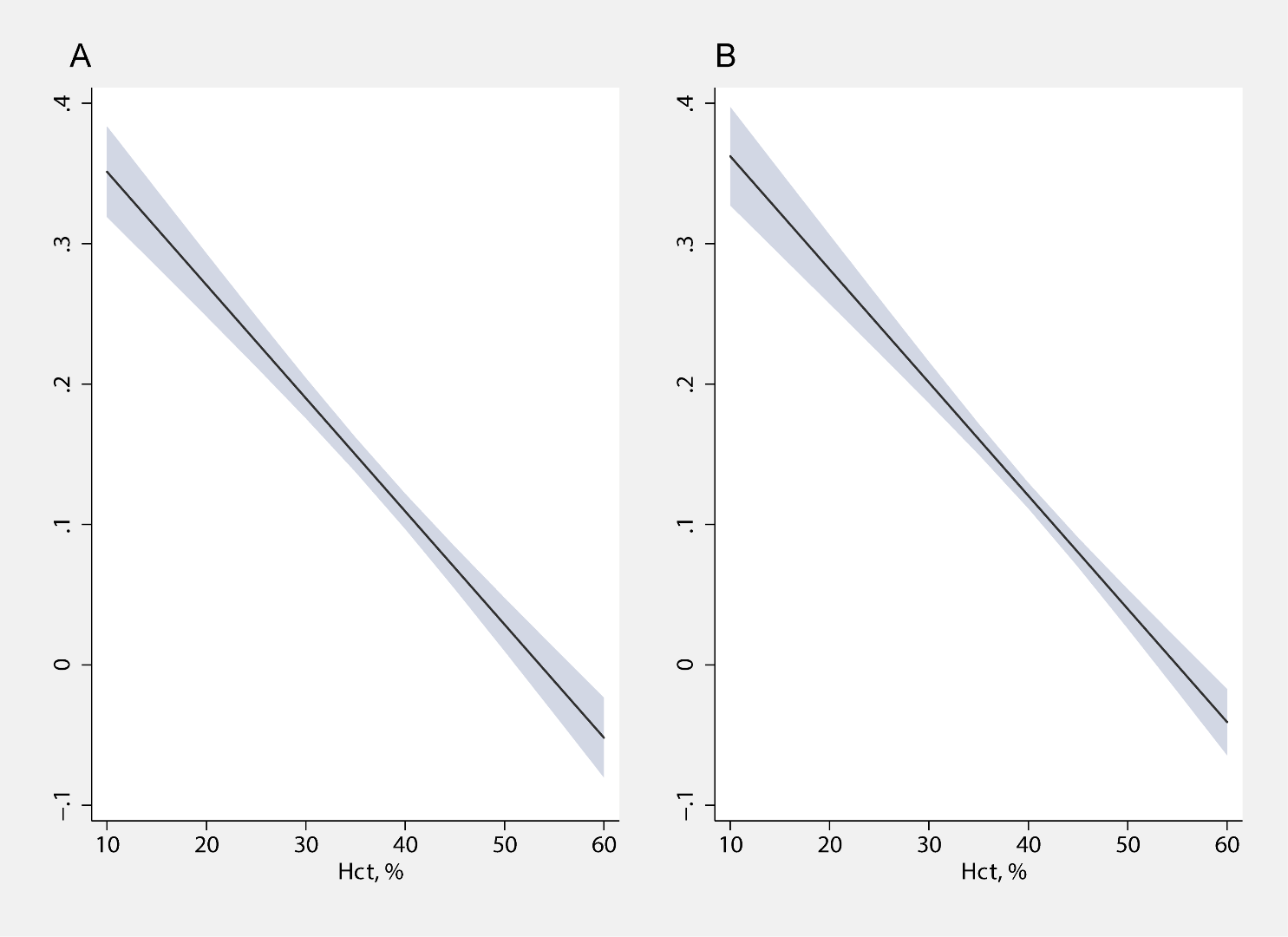


**Supplementary Fig. 3**. Subgroup analysis of sex on the relationship between HCT and AKI assessed by the marginal effect analysis. (**A**) in male patients; (**B**) in female patients.
